# Case Series of Recurring Spontaneous Closure of Macular Hole

**DOI:** 10.1155/2019/2398342

**Published:** 2019-06-16

**Authors:** Abdelrahman M. Elhusseiny, William E. Smiddy, Harry W. Flynn, Stephen G. Schwartz

**Affiliations:** ^1^Department of Ophthalmology, Bascom Palmer Eye Institute, University of Miami Miller School of Medicine, 900 NW 17 Street, Miami, FL 33136, USA; ^2^Department of Ophthalmology, Kasr Al Ainy School of Medicine, Cairo University, Egypt

## Abstract

Macular hole can undergo spontaneous reopening and reclosure. This is a retrospective review of three patients who had spontaneous reopening and reclosure of previously spontaneously closed macular hole documented by optical coherence tomography. We report the first case of nivolumab-uveitis-associated macular hole formation. The authors hypothesize that cystoid macular edema (CME) might alter the integrity of foveal tissues or conversely the orientation of the macular hole edges and play a role in formation and resolution of a macular hole.

## 1. Introduction

Macular holes (MH) are a common cause of decreased central vision. Most MHs are idiopathic, but secondary causes include trauma and uveitis. Surgery is usually recommended, but spontaneous closure of full thickness macular hole (FTMH) has been reported in 0-6% with one series as high as 11.5% [1].

In as much as the original pathogenesis is incompletely understood, the mechanisms of spontaneous closure and reopening are even less well understood. Certain subsets, as uveitis-induced MH, are more likely to close spontaneously and perhaps physical effects from cystoid macular edema play a role [2]. Conversely, macular edema per se, rather than inflammation, has been implicated as a causative element [3].

We present three patients who each had spontaneous MH closure, followed by reopening and reclosure; the MH closed spontaneously twice. One of these involved uveitis-associated macular edema caused by nivolumab therapy and represents the first reported case associated with MH formation.

## 2. Case Description

**Figure 1:**
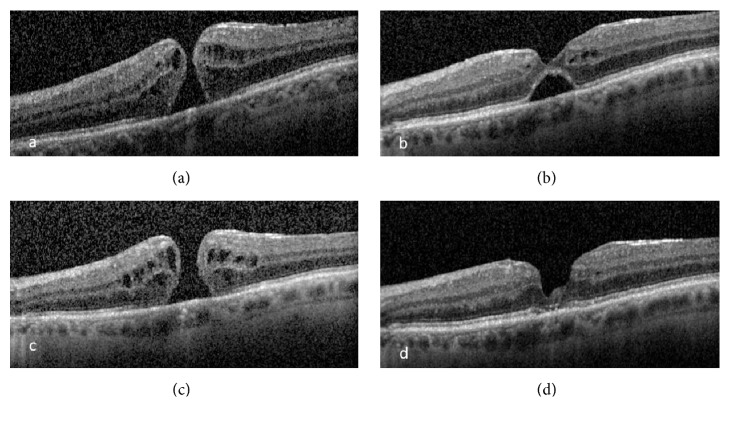
Optical coherence tomography showing (a) full thickness macular hole, (b) first closure of macular hole with residual subretinal fluid, (c) reopening of macular hole, and (d) second closure of the macular hole.

*Case 1*. An 80-year-old male presented with bilateral posterior uveitis and CME with onset 5 months after initiating nivolumab (Opdivo) for treatment of metastatic cancer due to an unknown primary tumor. Best corrected visual acuity (BCVA) was 20/70 OD and 20/40 OS. Oral prednisone, topical difluprednate (Durezol), and nepafenac (Nevanac) were started. CME had resolved with improved BCVA (20/30 OU) after 6 months of therapy. 9 months later, foveal thinning developed which progressed to a FTMH one month later (Figure 1(a)), reducing BCVA to 20/60 OS. Scheduled macular hole surgery (MHS) was cancelled when the vision improved to 20/40. OCT showed a closed MH, with residual subretinal fluid (SRF) (Figure 1(b)). The condition remained stable until three months later when the patient presented with decreased VA (20/150). OCT showed reopening of the MH (Figure 1(c)). The patient scheduled MHS but wanted to wait for 3 months, hoping for spontaneous resolution. 3 months later, MH spontaneously closed (Figure 1(d)) with improved VA to 20/80 OS. The condition has remained stable with 20/70 BCVA.

**Figure 2:**
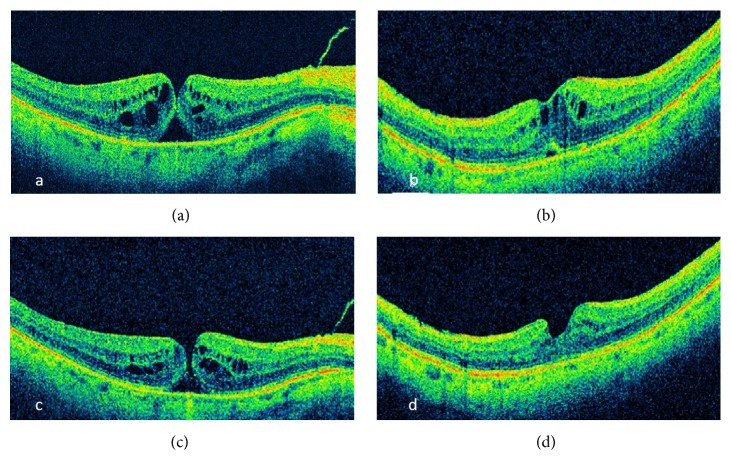
Optical coherence tomography showing (a) tiny full thickness macular hole, (b) first closure of macular hole, (c) reopening of macular hole, and (d) reclosure of macular hole.

*Case 2*. A 73-year-old male patient presented with a 2-month history of decreased vision OD (20/60). OCT showed a thinned fovea, progressing over 2 weeks into a tiny MH (Figure 2(a)). The patient was counseled about the treatment options and MHS was scheduled. 6 weeks later, vision improved (20/50), and OCT showed a closed MH although with residual CME (Figure 2(b)). 3 months later, MH had reopened (Figure 2(c)) with decreased vision (20/70), but the patient deferred MHS. Over 3 months, MH gradually reapproximated and closed with residual intraretinal CME and SRF which resolved slowly over 6 months (Figure 2(d)) with improved BCVA to 20/50 and remained stable during the next 7 months.

**Figure 3:**
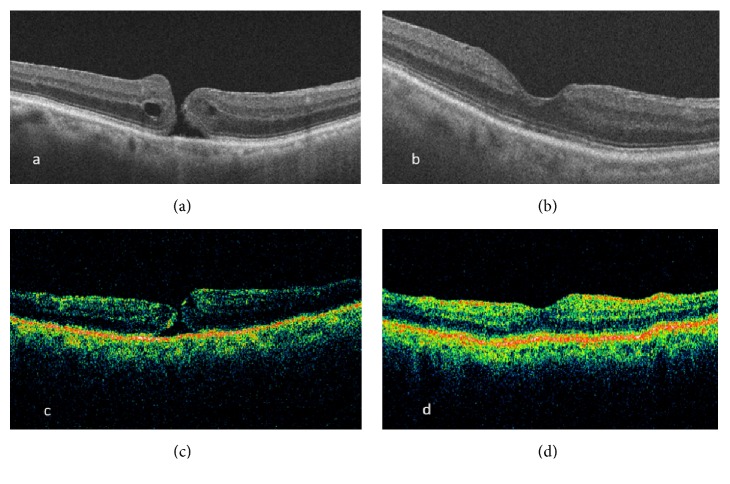
Optical coherence tomography showing (a) full thickness macular hole, (b) first closure of the macular hole, (c) reopening of the macular hole, and (d) second closure of macular hole.

*Case 3*. An 85-year-old female patient with a history of pseudoexfoliative glaucoma presented with a 4-month history of decreased vision OD (20/60). OCT showed MH (Figure 3(a)). 1 month later, spontaneous closure of the MH was observed (Figure 2(b)) with BCVA of 20/50. After 3 years, patient complained of a central scotoma OD. BCVA was 20/70. OCT showed a MH with intraretinal cystic spaces (Figure 3(c)). MHS was recommended but the patient declined. 1 month later, the MH had again spontaneously closed with a few cystic spaces and minimal SRF which resolved gradually (Figure 3(d)) although the BCVA was 20/125.

## 3. Conclusions

These three cases represent an unusual occurrence in which a macular hole spontaneously closed, opened, and then closed again. Spontaneous closure of a MH has been widely reported [4]. Also reopening of a previously surgically or spontaneously closed MH has been described [5]. A case with multiple spontaneous opening and closure of a myopic MH was reported [6]. Nivolumab-associated uveitis (present in Case 1) has only rarely been reported to cause CME but has never been reported in association with a MH.

The mechanism of spontaneous MH closure is unknown, but several hypotheses have been reported. OCT findings suggest that relief of anteroposterior traction after posterior hyaloid separation results in flattening of the MH edges [7]. This may then allow cells to bridge the hole and result in spontaneous MH closure. A postmortem histopathologic study suggested exposed retinal pigment epithelial (RPE) cells play a role by migrating onto the inner retinal surface, proliferate and contract circumferentially, pulling MH edges centripetally to facilitate spontaneous closure [8]. This seemed to be consistent with a clinical case report [5]. The normal accumulation of intraretinal fluid is a characteristic, but variably severe, feature of early MH formation; we hypothesize that frank CME might facilitate spontaneous closure by reapproximating the edges through physical expansion, a sort of healing by primary intention. Intercurrent CME, as in uveitis or otherwise, might exercise this effect. CME seemed to have been present in this way in case 2 when the spontaneous closure occurred.

Also, the mechanism of MH reopening is not understood. Chronic or recurrent CME might play a (different) role in some cases by weakening retinal tissue (particularly Muller cells). This may cause progressive retinal attenuation or render degenerated retina more vulnerable to incidental retinal surface or vitreous traction [3]. Gass proposed that rupture of the cystic spaces inner wall resulted in collapse of the cyst with subsequent retraction of the adjacent retinal tissue forming lamellar macular hole [9], so potentially a full thickness defect could similarly result. Uveitis has been reported to be associated with MH formation through either CME or chorioretinitis-induced structural weakening, vitreous liquefaction causing anomalous vitreomacular adhesions, or ERM formation [2].

Nivolumab is a recently approved checkpoint inhibitor, a monoclonal antibody for treatment of metastatic melanoma, renal carcinoma, and treatment-resistant cancer. It binds to programmed cell death protein 1 receptors on T-lymphocytes to enhance the host immunity against neoplastic cells [10]. Activation of the immune system may lead to adverse events such as autoimmune uveitis, hepatitis, pneumonitis, and renal failure [10]. Rarely reported ocular adverse effects of nivolumab-induced inflammatory cytokines include uveitis, Vogt-Koyanagi-Harada like syndrome, dry eye, keratitis, and CME [10]. Nivolumab-induced uveitis with CME has been sparsely reported previously, but not in association with MH formation as in Case 1. Nivolumab and ipilimumab (Yervoy) in combination have been associated with improved survival in cancer patients; ipilimumab has also been reported to cause ocular and orbital inflammation but was not used in our patient.
